# Health assessment of rice cultivated and harvested from plasma-irradiated seeds

**DOI:** 10.1038/s41598-023-43897-y

**Published:** 2023-10-14

**Authors:** Takamasa Okumura, Hayate Tanaka, Takumi Nakao, Teruki Anan, Ryo Arita, Masaki Shiraki, Kayo Shiraki, Tomoyuki Miyabe, Daisuke Yamashita, Kayo Matsuo, Pankaj Attri, Kunihiro Kamataki, Naoto Yamashita, Naho Itagaki, Masaharu Shiratani, Satoshi Hosoda, Akiyo Tanaka, Yushi Ishibashi, Kazunori Koga

**Affiliations:** 1 Faculty of Information Science and Electrical Engineering, Kyushu University, Fukuoka, 819-0395 Japan; 2 Graduate School of Information Science and Electrical Engineering, Kyushu University, Fukuoka, 819-0395 Japan; 3 Wano BARU Co. Ltd, Ogori, Fukuoka, 838-0106 Japan; 4 Center of Plasma Nano-interface Engineering, Kyushu University, Fukuoka, 819-0395 Japan; 5 Japan Aerospace Exploration Agency, Sagamihara, Kanagawa 252-5210 Japan; 6 Faculty of Medical Sciences, Kyushu University, Fukuoka, 812-8582 Japan; 7 Faculty of Agriculture, Kyushu University, Fukuoka, 819-0395 Japan

**Keywords:** Plasma physics, Electrical and electronic engineering

## Abstract

This study provides the health effects assessment of rice cultivated from plasma-irradiated seeds. The rice (*Oryza sativa L.*) cultivated from seeds with plasma irradiation showed a growth improvement (slope-ratios of with plasma to without plasma were 1.066, 1.042, and 1.255 for tiller, and earing, and ripening periods, respectively) and an 4% increase in yield. The cultivated rice was used for repeated oral administrations to mice for 4-week period. Distilled water and rice cultivated from seeds without plasma irradiation were also used as control. The weights of the lung, kidney, liver, and spleen, with corresponding average values of 0.22 g, 0.72 g, 2.1 g, and 0.17 g for w/ plasma group and 0.22 g, 0.68 g, 2.16 g, and 0.14 g for w/o plasma group, respectively, showing no effect due to the administration of rice cultivated from plasma-irradiated seeds. Nutritional status, liver function, kidney function, and lipid, neutral fat profiles, and glucose metabolism have no significant difference between with and without plasma groups. These results show no obvious subacute effects were observed on rice grains cultivated and harvested from the mother plant that experienced growth improvement by plasma irradiation. This study provides a new finding that there is no apparent adverse health effect on the grains harvested from the plasma-irradiated seeds.

## Introduction

The growing world population is placing unprecedented demands on agriculture. Currently, there are limited ways to ensure a stable food supply for the growing population, such as increasing yields and reducing food losses^1^. The pressure on agriculture leads to deforestation to secure agricultural land area, irrigation for adjusting or extending the cropping calendar and using chemical fertilizers for increasing yields. Food production must grow significantly to meet the world’s food security and sustainability needs. Still, agriculture’s environmental footprint must be simultaneously shrunk. One such approach is to use atmospheric-pressure plasma.

The plasma can enhance seed germination and increase yield without requiring additional land, water, and fertilizers^2–4^. In particular, the energy consumption of plasma irradiation of seeds is negligible compared to other agricultural operations, and atmospheric pressure plasma irradiation is expected to increase yields in a low-cost and environmentally friendly method. To date, there have been pioneering studies on improving germination and growth^5–20^. In a previous study, dielectric barrier discharge (DBD) plasma irradiation for 3 min showed a 56% increase in yield and an 11% reduction in harvest time for *Arabidopsis thaliana* seeds^10^. DBD plasma also can control of hormone balance between gibberellic acid (GA) and abscisic acid (ABA) in seeds^21,22^, and improvement of harvest characteristics^23^. Recently, molecular biological studies were conducted to elucidate the mechanisms underlying the biological effects of plasma irradiation^24^. A quantitative evaluation method for the amount of reactive oxygen and nitrogen species (RONS) introduced in seeds is being established for quantitative discussion on the above biological effects^25^. The above results indicate that previous reports dealing with plasma irradiation to seeds have laid the groundwork for further research.

Evaluation of health effects and composition of products cultivated from plasma-irradiated seeds are important topics to realize the social implementation of plasma technology. According to the European Commission’s Guidance on Novel Foods designated by the EU, the scientific data should include information on nutrients and toxicity based on expected intakes For European Food Safety Authority to assess the safety of Novel Foods concerning this technology, the following two aspects are important, which are that the novel food is at least as safe as food produced from conventional methods and that the novel food does not nutritionally prejudice consumers when it replaces other foods. Therefore, a critical issue to the success the social implementation of plasma irradiation to seeds is to determine the health effects and composition of the harvested crops from the plasma-irradiated seeds. However, as far as the authors know, there are no reports in which the health effects on other organisms and composition of harvested crops from plasma-irradiated seeds have been investigated, despite the various clinical trials for the medical application of plasma^26–31^. Thus, this study aimed to evaluate fundamental information on the composition and health effects of a crop cultivated from plasma-irradiated seeds.

This paper is the first case study to evaluate the effects of foods that have experienced growth promotion by plasma irradiation on health. Risk analysis is a process that incorporates risk assessment, risk management, and risk communication^32,33^. One of the basic goals of risk assessment is to provide information regarding the basis of a risk management decision. Therefore, the risk assessment was performed primarily to provide insight into who makes decisions about how that risk should be managed. In this study, plasma-irradiated rice seeds were cultivated, and the subacute health effects on experimental animals were investigated by orally administrating the harvested rice for 4 weeks. Since rice is the staple food of 2.7 billion people, almost half of the world’s population^34,35^, increasing rice yield by plasma irradiation will be a crucial technology for food production increase without deteriorating the global environment. Oral administration experiments over the lifetime of mice, a chronic effects evaluation test for about two years, are considered to be the most suitable for the health effects assessment using plasma-irradiated rice mice. However, this time, as the first stage of health effect evaluation, we conducted a subacute effect study in which repeated oral administration was performed for 4 weeks using mice. This paper revealed that the crop cultivated in a paddy field from plasma-irradiated seeds does not adversely affect mice’s health.

## Results and discussions

### Growth and crop yield

**Figure 1:**
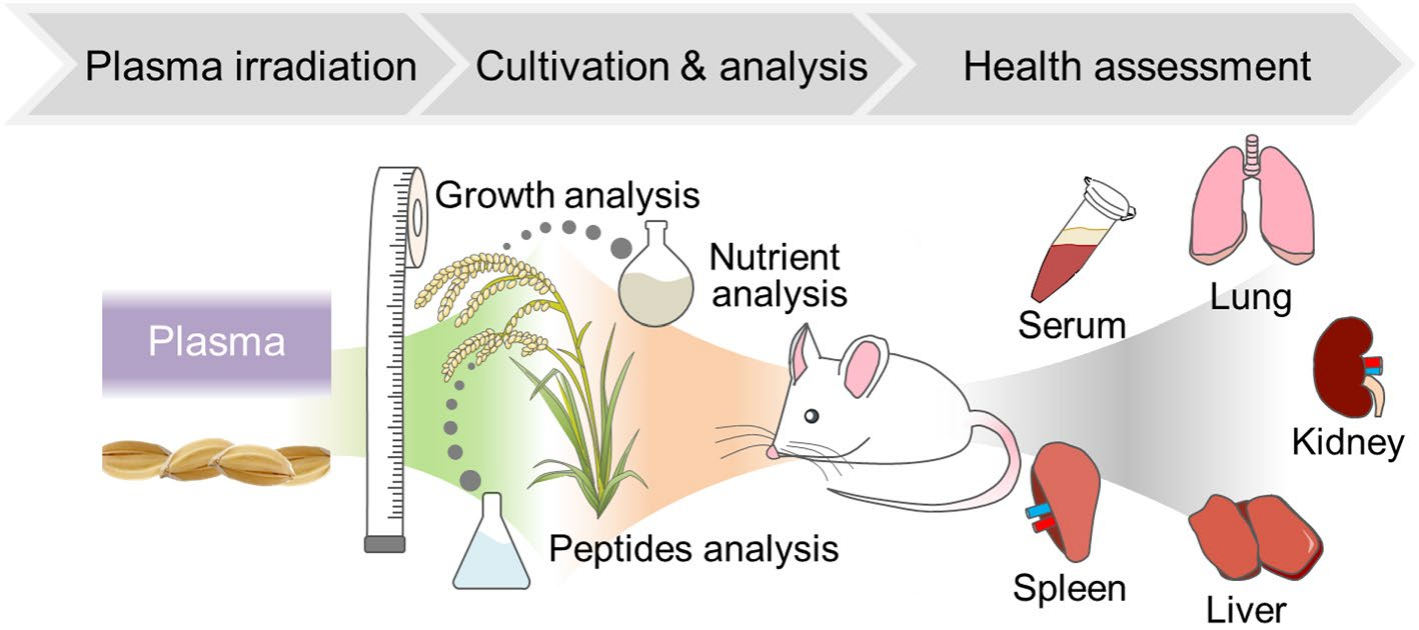
Graphical overview of experimental flow, created by the authors with reference to^36^.

**Figure 2:**
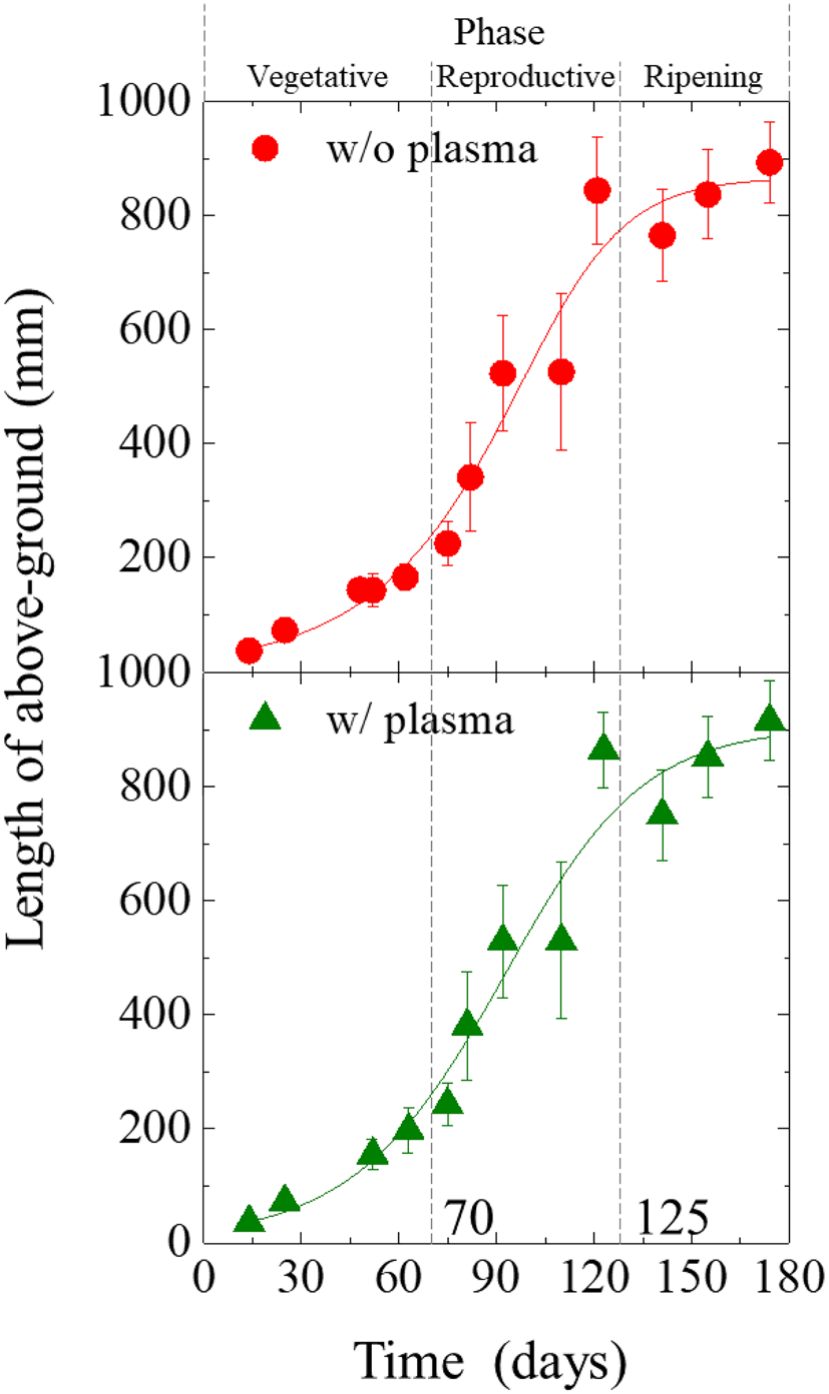
Length of above-ground of the samples without and with plasma irradiation as a function of cultivation time.

Figure 1 shows graphical overview of experimental flow, created by the authors with reference^36^. The seeds of rice (*Oryza sativa* L. cv. Hinohikari) were irradiated with DBD plasma for 3 min using a scalable DBD (SDBD) plasma device^10,22^. The discharge power density was 3.05 × 10^–2^ W mm^−2^. DBD produces RONS such as O(^1^D), *O_2_^–^, NO, NO_2_^–^, NO_3_^–^^25,37^. The plasma-irradiated rice seeds (w/plasma) and non-irradiated rice seeds (w/o plasma) were cultivated without fertilizers or pesticides in a paddy field in Ogori, Fukuoka. Figure 2 shows the length above-ground with time after seeding. Marks and error bars show the average values and standard deviations, respectively. In the early 70 days of growth period, the stalks' length linearly increased. From 70 to 125 days, tiller and earing period, the stalks length drastically increased. After 125 days, ripening period where the length does not change, the fruiting body matures instead. The slopes of growth, tiller, and earing, and ripening periods were calculated using the average values, and we obtained 3.10, 10.62, 1.48 mm/day for w/plasma group and 2.90, 10.20, 1.18 mm/day for w/o plasma group. These slope ratios of w/plasma to w/o plasma were 1.066, 1.042, and 1.255 for growth, tiller, and earing, and ripening periods, respectively, indicating that plasma irradiation to seeds increases the subsequent growth of rice. On the 170th day, the samples were harvested. The mean values and standard deviations of stalk length was 893.35 ± 69.63 mm in w/o plasma group and 915.32 ± 69.70 mm in seeds w/ plasma group (*p* = 0.00183). The stalks at the harvest showed about 2.5% higher in the plasma group compared to w/o plasma. The paddy weight obtained from the same field area of 4.6 × 10^–2^ ha was 181.98 and 189.29 kg for the control and plasma group, respectively. This indicates a 4% increase in yield by plasma irradiation. Consequently, plasma irradiation to seeds improves growth and increases crop yield. Such positive effects have been reported by Hashizume et al.^23^. We will conduct further research to elucidate the mechanism based on ensuring the reproducibility of field tests over multiple years. Assessment of the health effects of grains after harvesting plasma-irradiated rice seeds has great significance for the social implementation of these technologies.

### Chemical analysis of harvested rice

Chemical analysis for components of harvested rice were examined by the rice analysis service (Satake, Hiroshima, Japan) before the health effect assessment. We evaluated fundamentals in terms of moisture, ash, protein, fat, carbohydrates, and energy. The results shown in Table 1 suggest that moisture content, protein, and fat are slightly larger in w/plasma group than w/o plasma group. Ash and energy have no difference between w/o plasma and w/plasma groups. We also evaluated several peptides content as shown in Table 2. Although aspartic acid, glutamine, histidine, and arginine are relatively smaller in w/plasma group compared to w/o plasma group, serine, alanine, valine, and lysin suggest no difference between w/o plasma and w/plasma groups. Furthermore, there was no difference in the taste and appearance of the evaluation results obtained for steamed polished rice. However, the hardness was 4.06 and 3.86 kgf and the stickiness was 0.76 and 0.87 kgF, respectively, for w/o plasma and w/plasma, indicating that w/plasma group has the characteristic of absorbing and retaining water when steaming.

**Table 1:** Chemical analysis result of components of harvested rice in terms of water content, ash, protein, fat, carbohydrates, and energy obtained before the health effect assessment.

	Water content	Ash	Protein (mg/FWg)	Fat	Carbohydrates	Energy (kcal)
w/o plasma	149	13	71	21	746	349.6
w/o plasma	151	13	73	23	739	349.2

**Table 2:** Content of several peptides in harvested rice.

	Aspartic acid	Glutamine	Serine	Histidine	Glycine	Threonine	Arginine	Alanine
	Asp	Gln	Ser	His	Gly	Thr	Arg	Ala
w/o plasma	1.8	2.4	0.2	0.2	N.D	N.D	0.6	0.3
w/o plasma	1.6	2	0.2	0.1	N.D	N.D	0.5	0.3

	Gamma-aminobutylic acid	Tyrosine	Valine	Methionine	Phenylalanine	Isoleucine	Leucine	Lysine
	GABA	Tyr	Val	Met	Phe	Ile	Leu	Lys
w/o plasma	N.D	N.D	0.1	N.D	N.D	N.D	N.D	0.2
w/o plasma	N.D	N.D	0.1	N.D	N.D	N.D	N.D	0.2

All numeric values are in µg/FWg.

### Health effects assessment

**Figure 3:**
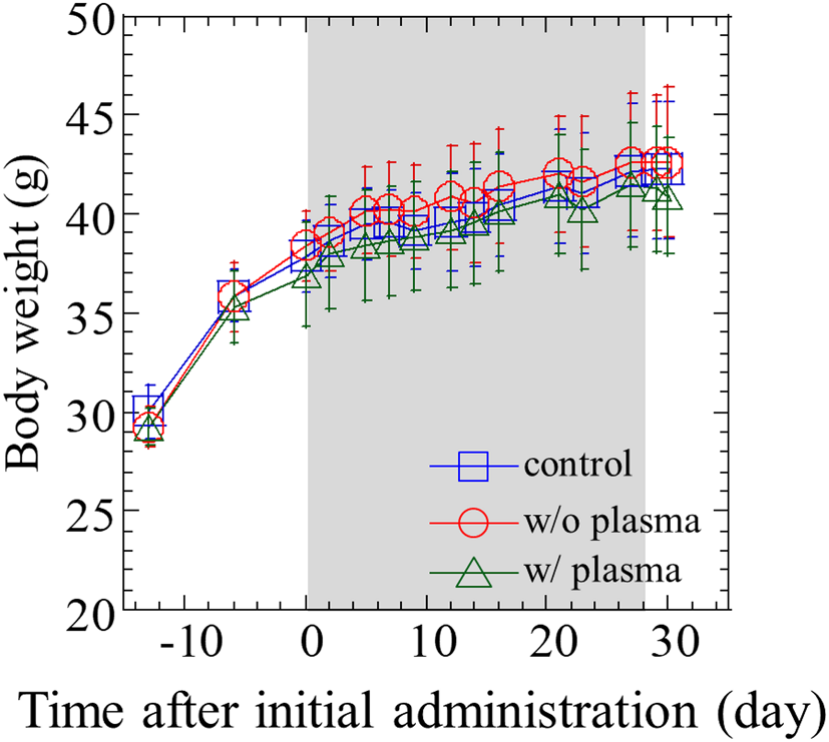
Body weight of mice with the time.

**Figure 4:**
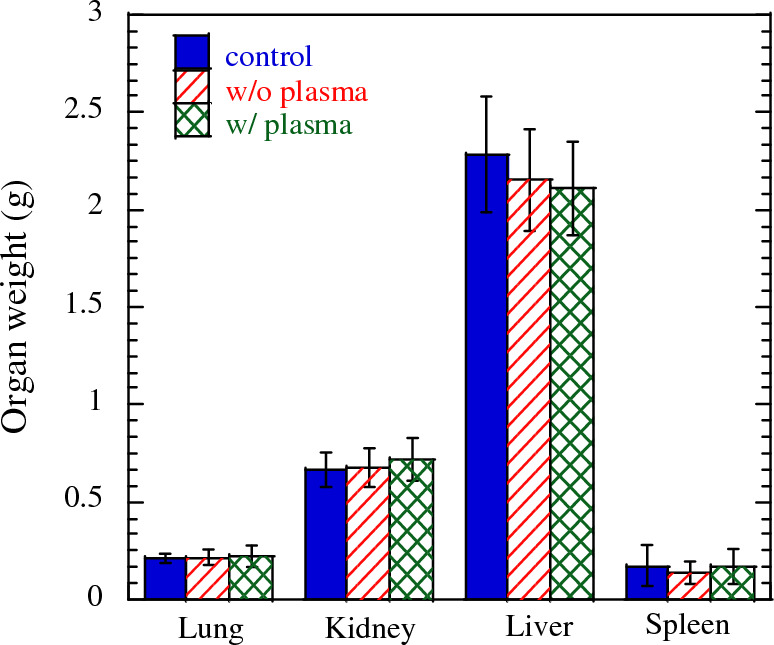
Weights of mice’s lung, liver, kidney, and spleen.

**Figure 5:**
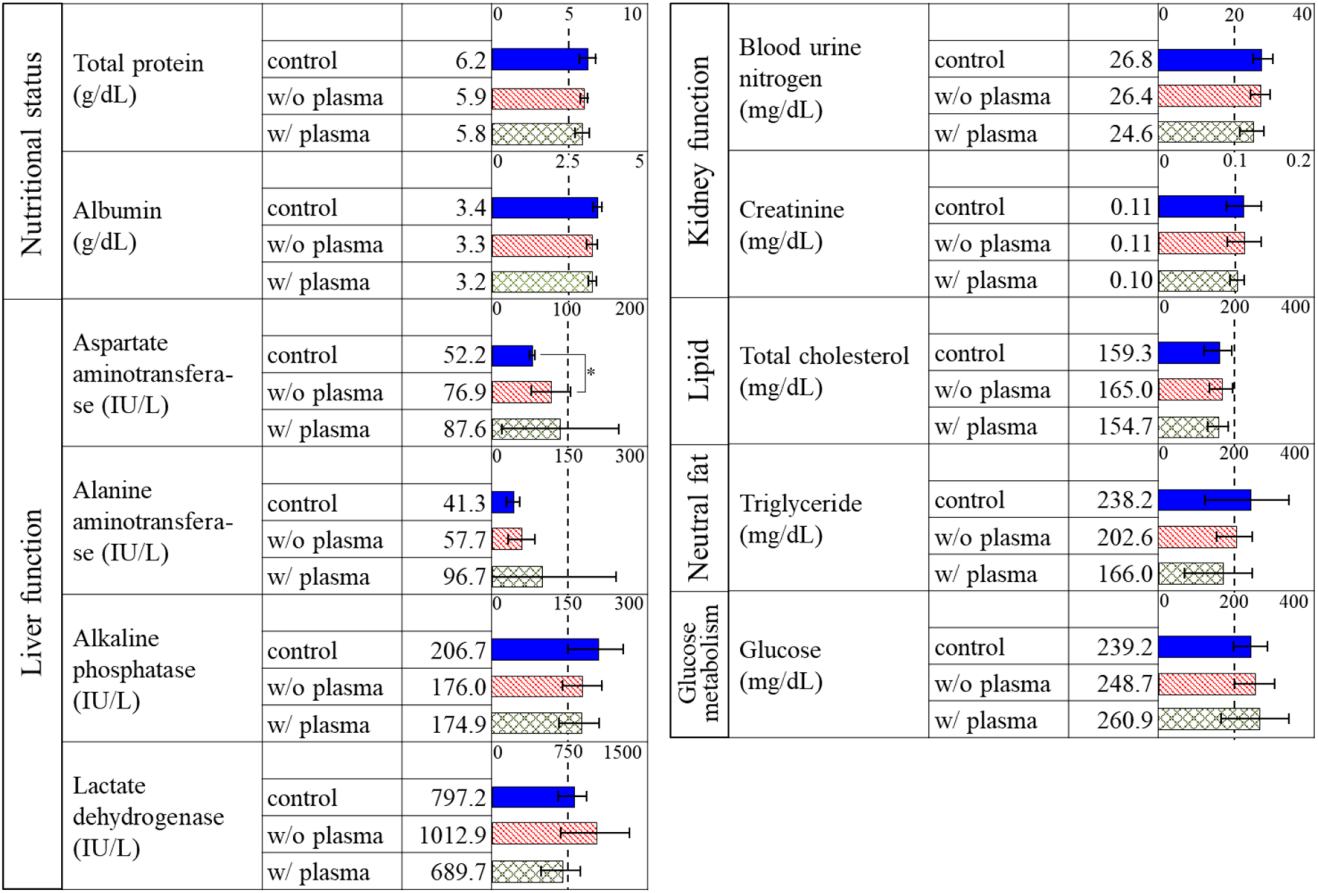
Health effects assessment by serum biochemistry.

The harvested rice was milled and administrated by oral dosing to mice. We prepared 3 administration groups; 1 ml/mouse of distilled water (control), 1 ml/mouse of 5% rice flour of w/o plasma or rice w/o plasma after autoclave. This amount corresponds to the rice consumed in a human adult’s diet. Instillation period was 3 times in a week for 4 weeks. At autopsy, one of 10 animals in the control group was observed to have an abscess in the left arachnoid paw, and one of 10 animals in the plasma-irradiated group had an abscess in the pelvic cavity, so they were excluded from the evaluation. Thus, 9 animals in the control group, 10 in the w/o plasma group, and 9 in the w/plasma group were included in the evaluation. Figure 3 shows the body weight of the mice with time since administration. The grey region in the figure indicates the period during the administration. The body weight increased with time. Slopes of the body weight are 0.14, 0.13, and 0.13 for control, w/o plasma, and w/ plasma, respectively, suggesting no significant difference. Moreover, no systemic symptoms or abnormal behavior were observed in each mouse and no significant differences in food intake in each group during the administration period. On the 28th day since the administration, mice were euthanized, and organs weight were measured. Figure 4 shows the weights of the lung, kidney, liver, and spleen, with corresponding average values of 0.22 g, 0.72 g, 2.1 g, and 0.17 g for w/ plasma group, 0.22 g, 0.68 g, 2.16 g, and 0.14 g for w/o plasma group, and 0.21 g, 0.67 g, 2.28 g, and 0.17 g for control group, respectively. This result showed no difference in group w/ plasma, compared to w/o plasma or control, indicating no effect due to the administration of rice cultivated from plasma-irradiated seeds on organ weights. Moreover, we evaluated the serum biochemical profiles. The results are shown in Fig. 5. We evaluated nutritional status, liver function, kidney function, lipid, neutral fat, and glucose metabolism profiles. Bars and error bars show the mean values and the standard deviations, respectively. The numbers next to the bars show the mean values. The nutritional status, in terms of total protein and albumin, has no significant difference among control, w/o plasma, and w/plasma group. For liver function, with respect to aspartate aminotransferase (AST) values, the values in w/o plasma group were significantly higher than those in the control group, but AST values in the plasma-irradiated group were not significantly different from those in control or w/o plasma groups. No significant differences in other liver function values, alanine aminotransferase (ALT), lactate dehydrogenase (LDH), and alkaline phosphatase (ALP), were observed among each groups. In the present study, an analysis of AST values revealed a significant increase in w/o plasma group in comparison to control group. In instances of liver damage, characterized by pronounced inflammation, a concomitant elevation in liver organ weight should be observed, however, no discernible disparity is noted in the liver organ weight between w/o plasma group and control group. Furthermore, an assessment of other indices associated with liver damage, such as ALT levels, did not reveal any statistically significant distinctions between w/o plasma group and control group. These findings collectively suggest the potential existence of mild liver damage, albeit the precise magnitude of this damage remains indeterminate. In Fig. 5, conspicuous deviations are observed in the levels of w/ and w/o plasma groups for ALT and AST when compared to control group. These deviations can be primarily attributed to the presence of outliers within the data, specifically, two mice in w/o plasma group and one mouse in w/plasma group exhibited AST values approximately an order of magnitude higher than the remaining subjects in their respective groups (see Supp. Table 1). Despite the pronounced variations in ALT and AST values stemming from these outlier mice, it is noteworthy that their kidney weights displayed no overt alterations. Consequently, the calculations for ALT and AST levels were performed while taking into consideration these atypical observations. For kidney function, blood urine nitrogen and creatinine show no significant difference. Total cholesterol as lipid profile was the same level for all groups. Comparing the mean values of triglyceride as a neutral fat profile, control, w/o plasma, and w/plasma group show 238.2, 202.6, and 166.0, respectively, but these differences show no significance. Glucose as a glucose metabolism profile showed no significant difference. These results show no subacute effects were observed on rice grains cultivated and harvested from the mother plant that experienced growth improvement by plasma irradiation. Although plasma irradiation does not require additional fertilizers or work in cultivation, there are numeral reports of improvements in growth and yield due to plasma irradiation^2–4^. This study provides a new finding that there is no clear adverse health effect on the grains harvested from the plasma-irradiated seeds.

## Conclusion

This study was conducted to evaluate whether a crop cultivated from plasma-irradiated seeds has a health effect. The plasma-irradiated rice seeds showed positive effects for growth enhancement during cultivation. The above-ground length and yield at the harvest was 2.5% and 4% higher in the plasma group compared to w/o plasma group, respectively. The chemical analysis of the harvested rice revealed that plasma irradiation exerts no large difference in fundamental characteristics and peptides. Health assessment revealed no obvious health effect in the dose range was found in terms of body weight, lung, liver, kidney, or spleen weight, serum biochemistry for ICR mice that were orally administrated with brawn rice cultivated from plasma-irradiated seeds, even the plasma-irradiation had improved the growth and increased the yield in the dose range. Consequently, it has been shown that the method of increasing the production of agricultural products by plasma irradiation is one of the safe methods without health effects.

## Material and methods

### Experimental set-up

Scalable dielectric barrier discharge (SDBD) device consisted of 20 stainless rod electrodes of 1 mm in outer diameter and 60 mm in length covered with a ceramic tube of 2 mm in an outer diameter. The electrodes were arranged parallel with a spacing of 0.2 mm. DBD plasma discharge was generated between the electrodes by supplying voltage of 7.0 kV with a frequency of 14.4 kHz using power supply (Logy Electric, LHV-09 K). The discharge power density in the air was 3.05 × 10^–2^ W mm^−2^. The temperature and relative humidity was 24 °C and 40–60%, respectively. 10 g of seeds were placed at 3 mm below SDBD electrode and irradiated with plasma for 3 min. We finally obtained 1 kg of seeds.

### Cultivation and harvest

The plant material was provided from Wano BARU Co., Fukuoka, Japan and used for experiments with their permission. The irradiated seeds were sterilized underwater at 60 °C, vernalized underwater at 25 °C, and sown in a paddy field. Then planted in a rice field and cultivated. 5814 strains were planted in fields with the same area. The paddy was harvested at each growth stage and evaluated the above-ground length. The cultivation was conducted in Fukuoka Prefecture in 2018, using homemade seeds produced in 2017. The cultivation was carried out without any chemical fertilizers or pesticides. All methods were performed in accordance with the relevant guidelines and regulations.

### Health assessment

Thirty male ICR mice from the colony of Japan SLC Inc. (Hamamatsu, Shizuoka, Japan) were purchased at six-week-old and housed under temperature conditions of between 22 ℃ and 25℃. Mice were maintained under a cycle of 12-h lighting, within a specific pathogen-free (SPF) conditions at the Laboratory of Animal Experiments, Graduate School of Medical Sciences, Kyushu University. Mice were given a commercial diet and water ad libitum. Repeated oral administrations were performed on 8-week-old mice after an acclimatization period of 2 weeks. These experiments were conducted according to the Regulations for Animal Experiments at Kyushu University and under the Law (No. 105) and Notification of the Government of Japan and performed in compliance with the ARRIVE guidelines. Rice was crushed finely, autoclaved, and then orally administered to mice. 5% rice flour was orally administered to 1 ml/mouse; each mouse's dose was calculated to be 1.25 g/kg BW. This is equivalent to 75 g intake for humans, assuming a human body weight of 60 kg. The oral administration was performed 3 times in a week for 4 weeks and thus a total 12 oral administrations. Body weight was measured at each administration. The day after the final administration, we evaluated body weight, organ weight, and serum biochemistry of 9 mice fed rice cultivated from plasma-irradiated seeds (these mice were called as with (w/) plasma), 10 mice fed rice cultivated from no plasma-irradiated seeds (without (w/o) plasma), and 9 mice fed just distilled water at the administrations (control). In all the statistical comparisons, a *p*-value of less than 0.05 was used to determine significant difference. A one-way analysis of variance was performed for items for which the Bartlett test did not reject the homogeneity of variance. The Kruskal–Wallis test was performed for items for which the homoscedasticity was rejected.

### Animal experiment approval

All experiments were conducted according to the regulations and approval for Animal Experiments at Kyushu University (A19-092-0) and under the Law (No. 105) and Notification of the Government of Japan and performed in compliance with the ARRIVE guidelines.

### Supplementary Information

Supplementary Table S1.

## Data Availability

The datasets used and/or analysed during the current study available from the corresponding author on reasonable request.
