# Supplementary material for: Health assessment of rice cultivated and harvested from plasma-irradiated seeds
Source: Sci Rep. 2023 Oct 14;13:17450. doi: 10.1038/s41598-023-43897-y (PMC10576742; doi:10.1038/s41598-023-43897-y)
Supplement: Supplementary file 1 — Supplementary Table S1. [file 41598_2023_43897_MOESM1_ESM.docx]

Supplementary information

Health Assessment of Rice Cultivated and Harvested from Plasma-irradiated Seeds

*Takamasa Okumura ^1^, Hayate Tanaka ^2^, Takumi Nakao ^3^, Teruki Anan ^4^, Ryo Arita ^5^, Masaki Shiraki ^6^, Kayo Shiraki ^7^, Tomoyuki Miyabe ^8^, Daisuke Yamashita ^9^, Kayo Matsuo ^10^, Pankaj Attri ^11^, Kunihiro Kamataki ^12^, Naoto Yamashita ^13^, Naho Itagaki ^14^, Masaharu Shiratani ^15^, Satoshi Hosoda ^16^, Akiyo Tanaka ^17^, Yushi Ishibashi ^18^, *Kazunori Koga ^19^

^1^ Faculty of Information Science and Electrical Engineering, Kyushu University, Fukuoka 819-0395, Japan

^2^ Graduate School of Information Science and Electrical Engineering, Kyushu University, Fukuoka 819-0395, Japan

^3^ Graduate School of Information Science and Electrical Engineering, Kyushu University, Fukuoka 819-0395, Japan

^4^ Graduate School of Information Science and Electrical Engineering, Kyushu University, Fukuoka 819-0395, Japan

^5^ Graduate School of Information Science and Electrical Engineering, Kyushu University, Fukuoka 819-0395, Japan

^6^ Wano BARU Co. Ltd., Fukuoka Japan

^7^ Wano BARU Co. Ltd., Fukuoka Japan

^8^ Wano BARU Co. Ltd., Fukuoka Japan

^9^ Faculty of Information Science and Electrical Engineering, Kyushu University, Fukuoka 819-0395, Japan

^10^ Faculty of Information Science and Electrical Engineering, Kyushu University, Fukuoka 819-0395, Japan

^11^ Center of Plasma Nano-interface Engineering, Kyushu University, Fukuoka 819-0395

^12^ Faculty of Information Science and Electrical Engineering, Kyushu University, Fukuoka 819-0395, Japan

^13^ Faculty of Information Science and Electrical Engineering, Kyushu University, Fukuoka 819-0395, Japan

^14^ Faculty of Information Science and Electrical Engineering, Kyushu University, Fukuoka 819-0395, Japan

^15^ Faculty of Information Science and Electrical Engineering, Kyushu University, Fukuoka 819-0395, Japan

^16^ Japan Aerospace Exploration Agency, Kanagawa 252-5210, Japan

^17^ Faculty of Medical Sciences, Kyushu University, Fukuoka 812-8582, Japan

^18^ Faculty of Agriculture, Kyushu University, Fukuoka 819-0395, Japan

^19^ Faculty of Information Science and Electrical Engineering, Kyushu University, Fukuoka 819-0395, Japan

E-mail: t.okumura@plasma.ed.kyushu-u.ac.jp, koga@ed.kyushu-u.ac.jp

**Table Captions for supporting files**

**Table S1**. Raw data of health effects assessment results by serum biochemistry for (a) control, (b) without plasma irradiation, (c) with plasma irradiation.


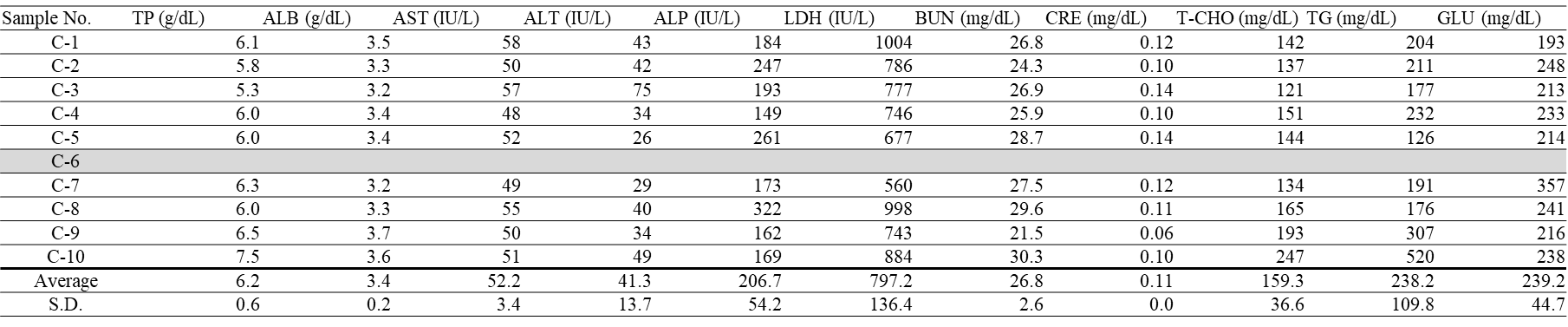
 **
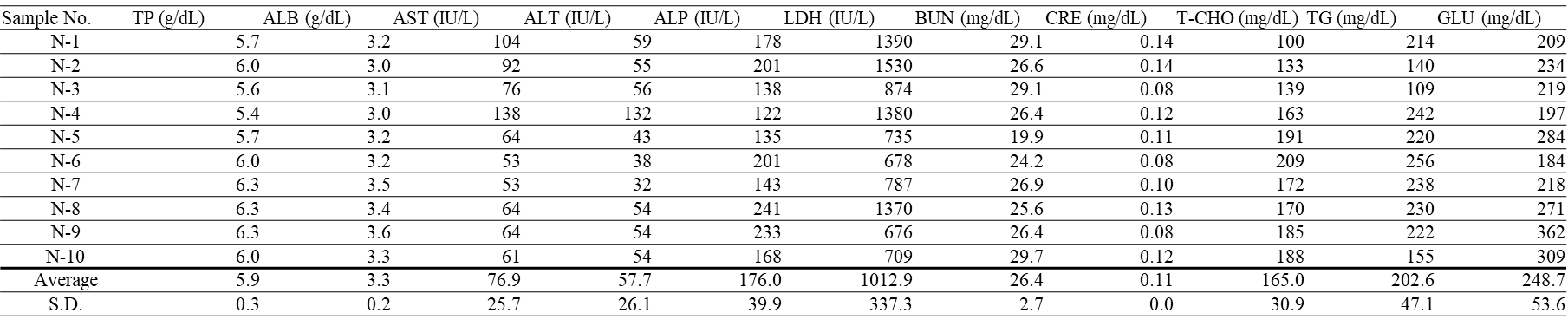

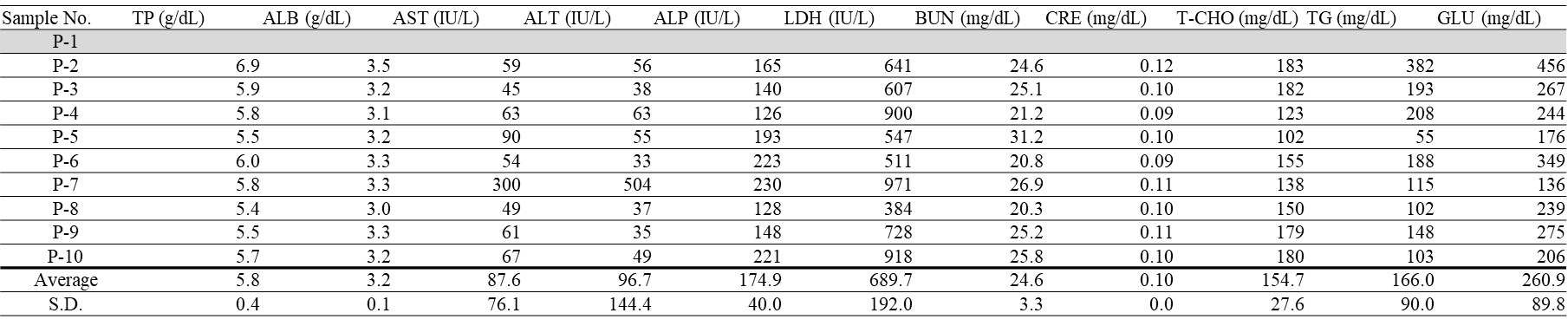
**

**Table S1. (c)**

**Table S1. (b)**

**Table S1. (a)**
